# Retraction: Myogenic differentiation of VCP disease-induced pluripotent stem cells: A novel platform for drug discovery

**DOI:** 10.1371/journal.pone.0347383

**Published:** 2026-04-16

**Authors:** The PLOS One Editors

After this article [1] was published, concerns were raised regarding results presented in Figs 1, 4, and 5.

Specifically:

- In Fig 1B, the upper-right of the Patient Day 50 panel appears discontinuous with the surrounding image areas.
- Fig 1C and Fig 1E appear to partially overlap.
- The Fig 4E Perifosine LC3 panel appears similar to the Fig 5B Patient Untreated LC3 panel.
- In Fig 5F LC3I/II panel, there appears to be a vertical discontinuity between lanes 6 and 7.

The corresponding author stated that an error occurred in the assembly of the Fig 1B Patient Day 50 panel. They provided the original underlying image for this panel and PLOS considers this concern resolved.

The corresponding author stated that the Fig 1E and Fig 5B Untreated LC3 panels are incorrect and provided replacement images and underlying image data for these panels. Upon editorial assessment, PLOS identified additional concerns for the underlying data and replacement panel provided for Fig 5B that call into question the reliability of the reported results in this figure.

The corresponding author stated that the Fig 5F LC3I/II panel was assembled from two different blots because lanes 7–8 were not visible in the original blot, and that these lanes in Fig 5F in [1] were obtained from a repeat blot. They provided the underlying blot for TDP-43 in Fig 4G and the underlying blot for lanes 1–6 in Fig 5F and stated the underlying blot for lanes 7–8 in Fig 5F are no longer available. The authors’ explanation calls into question the reliability of the associated quantified results in Fig 5G, which cannot be verified in the absence of the original blot data. Upon editorial assessment the *PLOS One* Editors noted additional concerns for the underlying blot data provided for Figs 4G and 5F, raising further concerns for the reliability of these figures in [1].

In light of the above unresolved concerns that question the reliability and integrity of the reported results, the *PLOS One* Editors retract this article.

BT did not agree with the retraction. LNW, IC, and VEK did not agree with the retraction, stand by the article’s findings and apologize for the issues with the published article. BK did not agree with the retraction and stands by the article’s findings. HY did not agree with the retraction and apologizes for the issues with the published article. KJL, AN, and VS either did not respond directly or could not be reached.

In addition to the concerns above, the *PLOS One* Editors are aware of similarities between results presented in Fig 2F FACS panels in [1] and results presented in Figs 2A and 2B FACS panels in a later article by different authors [2].
